# Temporal compounding increases economic impacts of atmospheric rivers in California

**DOI:** 10.1126/sciadv.adi7905

**Published:** 2024-01-19

**Authors:** Corinne Bowers, Katherine A. Serafin, Jack W. Baker

**Affiliations:** ^1^ Civil and Environmental Engineering Department, Stanford University, Stanford, CA 94304, USA.; ^2^ Department of Geography, University of Florida, Gainesville, FL 32611, USA.

## Abstract

Temporally compounding atmospheric river (AR) events cause severe flooding and damage in California. However, the contribution of temporal compounding to AR-induced loss has yet to be systematically quantified. We show that the strongest ARs are more likely to be part of sequences, which are periods of elevated hydrologic hazard associated with temporally clustered ARs. Sequences increase the likelihood of flood-related impacts by 8.3% on AR days and 5.4% on non-AR days, and across two independent loss datasets, we find that ARs within sequences have over three times higher expected losses compared to ARs outside of sequences. Expected losses also increase when the preceding AR is higher intensity, when time since the preceding AR is shorter, and when an AR is the second or later event within a sequence. We conclude that temporal compounding is a critical source of information for predicting an AR’s potential consequences.

## Teaser

Sequences of back-to-back atmospheric rivers increase likelihood and magnitude of flood damage compared to individual events.

## INTRODUCTION

Atmospheric rivers (ARs) are the primary drivers of hydrologic extremes and flood loss in California and the western United States. These elongated corridors of high moisture flux can deliver up to half of California’s annual water supply (*1*), making ARs an essential component of the state’s highly variable hydroclimate. However, while ARs can alleviate water scarcity issues, they are both globally and regionally associated with extreme precipitation (*2*, *3*) and hydrologic floods (*4*–*8*). Climate change is projected to increase the frequency of the most extreme ARs at the expense of more moderate ones (*9*–*12*), intensifying the potential severity of future consequences (*13*).

In addition to hydrologic impacts, ARs cause economic impacts through flood damage and flood loss, totaling approximately $1.1 billion for the western US and $620 million for California in 2019 dollars annually (*14*). To date, most attempts to estimate economic losses resulting from AR-driven flooding have relied on the Ralph *et al.* (*15*) intensity scale, which assigns each event an intensity rank from AR1 to AR5 based on the maximum integrated water vapor transport (IVT; kilogram per meter per second) and the duration (hours) of IVT exceeding 250 kg m^−1^ s^−1^. Corringham *et al.* (*14*) developed a relationship between AR intensity rank and estimated loss that has been used extensively by others to quantify AR impacts in the present (*16*) and future (*17*, *18*). However, while Corringham *et al.* (*14*) showed that median losses increase by about an order of magnitude with increasing AR intensity, the 90% confidence interval (CI) around each median was three to four orders of magnitude. This illustrates that while intensity is an important predictor of losses, there is still incredible variance in loss outcomes between events of the same rank, and substantial uncertainty remains in our understanding of an AR’s potential for economic consequences. Additional factors that influence AR impacts and potentially contribute to this uncertainty include AR orientation (*19*, *20*), antecedent soil moisture and/or hydrologic conditions (*21*, *22*), freezing level (*23*, *24*), and the limitations of existing flood control management strategies (*25*, *26*), to name a few.

The role of temporal compounding, though, has received comparably less attention. Temporal compounding, one of the four types of compounding identified by Zscheischler *et al.* (*27*), is the amplified risk that results from multiple hazard events occurring in close succession. In the context of ARs, temporal compounding increases risk because earlier events can raise the soil moisture or snowpack levels and set up conditions so that following events cause greater flooding. This phenomenon has particular relevance for California, where almost all AR activity is compressed into the 6-month wet season and storms are more likely to occur in clusters (*28*). The state most recently experienced the disastrous effects of temporal compounding in the first few weeks of 2023, when a series of nine ARs occurred in rapid succession (*29*) and caused record-setting precipitation and widespread flooding in communities across both northern and southern California (*30*). The overwhelming statewide impacts led to a Presidential Major Disaster Declaration on 14 January, and preliminary estimates of total losses exceed 3 billion dollars (*31*). Before that, the 2017 Oroville Dam crisis that forced the evacuation of nearly 200,000 downstream residents was due in part to the compounding effects of a 2-month series of strong AR events (*32*, *33*). Huang and Swain (*34*) have further shown that AR “megafloods,” which have occurred in California’s past and are projected to occur more frequently due to climate change, are likely to come from long-duration sequences of storms rather than individual AR events.

There is therefore a growing need to both (i) identify when temporal compounding is contributing to AR impacts and (ii) quantify how compounding intensifies those impacts relative to the expected consequences of individual AR events. Bowers (*35*) addressed the first of these two needs by proposing a definition of AR sequences based on moving average IVT. Sequences define the time windows of clustered AR activity where temporal compounding is contributing to amplified risk of flooding and negative consequences. Additional definitions of temporal compounding include AR families (*36*, *37*), which are defined based on a set aggregation period and have been linked to damaging ARs originating in the West Pacific (*16*). However, most of the work on AR families is concerned with differences in the synoptic environments between families and individual ARs. Sequences, on the other hand, focus directly on impacts and have been shown to capture time windows of high hydrologic hazard, namely, elevated runoff and soil moisture (*35*).

This paper addresses the second need by quantifying the economic impacts associated with AR sequences. The results are divided into five sections. The first section explores the relationship between temporal compounding and AR intensity. ARs are identified on a 50-km grid using the Rutz *et al.* (*38*) detection algorithm and assigned a Ralph *et al.* (*15*) intensity rank. We then combine information about ARs, sequences, and temporal compounding with two datasets measuring impact and loss: the National Centers for Environmental Information (NCEI) Storm Events Database (*39*) and the National Flood Insurance Program (NFIP) claims database (*40*). The second section calculates the effect of sequences on the probability of impact, where impact is defined as any day where either the NCEI database records a hydrologic event or there is at least one NFIP claim in the grid cell (this includes zero-loss events and claims). The NCEI database measures storm-related event total losses at the county level and is overall more geographically complete, with particularly high representation of severe winter weather events on the windward slopes of the Sierra Nevada mountains. NFIP claims measure property-level insured flood losses, mostly to residential structures, and are heavily concentrated in populated coastal areas. This work directly compares estimated flood costs between these two separate datasets at the event scale in California.

The third section calculates the effect of sequences on the magnitude of loss, where losses are defined when either the NCEI database records a hydrologic event with a nonzero loss total or the NFIP reports a nonzero claim. Loss results are presented separately for the two databases. The fourth section explores how the strength of the preceding AR and time since the preceding AR affect the current AR, and the fifth section estimates the loss associated with earlier versus later ARs in a sequence. We conclude by advocating for evaluating ARs through a broader lens than an individual event perspective to assess, understand, and predict AR impacts.

## RESULTS

### Temporal compounding and AR magnitude

**Fig. 1.**
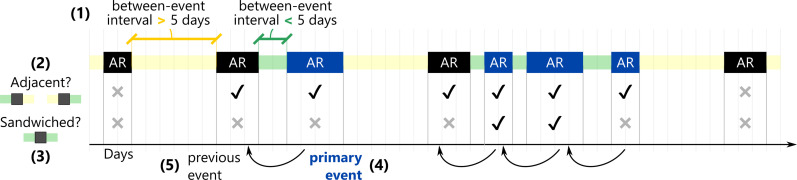
Schematic of terms used to describe temporal relationships: Between-event interval, adjacent, sandwiched, primary event, and previous event. Between-event intervals are used in Fig. 2 and 6, adjacent and sandwiched ARs are used in Fig. 2, and previous and primary events are used in Fig. 3 and 6. (1) The “between-event interval” is the time, in days, from the end of the last AR event to the start of the next one. In this schematic, intervals less than 5 days are colored in green, and intervals greater than 5 days are colored in yellow. (2) “Adjacent” ARs are those where the between-event interval before or after is less than 5 days, i.e., either the preceding or the following between-event interval is green. (3) “Sandwiched” ARs are those where the between-event intervals both before and after are less than 5 days, i.e., both the preceding and following between-event intervals are green. (4 and 5) To define primary and previous events, we focus only on the subset of ARs in blue that have preceding between-event intervals of less than 5 days. For each of these four events, the “primary event” is the AR in blue (start of the arrow), and the “previous event” is the AR immediately preceding it (end of the arrow).

**Fig. 2.**
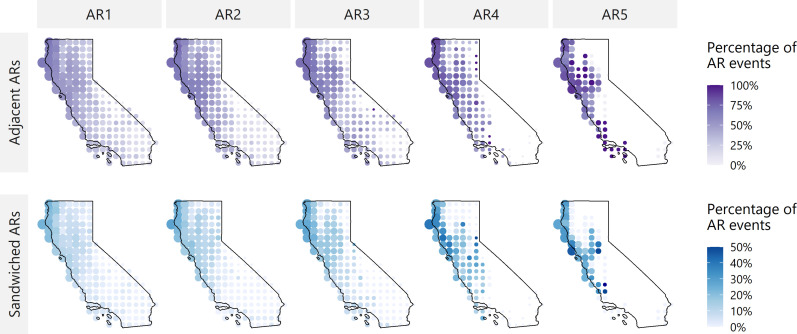
Probability of another AR within ±5 days. The columns represent AR intensity ranks AR1 to AR5, and the rows represent the probability of an AR occurring before or after, referred to as the adjacent case (top) and the probability of an AR occurring before and after, referred to as the sandwiched case (bottom). Color indicates the probability of a given AR event in that location being adjacent or sandwiched. Size of the bubble indicates the frequency of ARs of a given magnitude relative to the statewide total number of ARs of that magnitude from WY 1981 to 2021: AR1 = 21,078, AR2 = 10,358, AR3 = 4812, AR4 = 1230, and AR5 = 323. In both rows, the probability of an AR being adjacent and/or sandwiched increases moving from south to north and moving from smaller to larger magnitudes, indicating that more intense ARs are more likely to occur in close temporal proximity to other AR events.

The first set of analyses examines whether higher-intensity ARs are more likely than lower-intensity ones to occur in close temporal proximity to other AR events. We compute the probability of AR events occurring within a specified time window, using a 5-day interval between events as the metric of proximity (*35*, *36*), and define two types of compounding as follows: “Adjacent” ARs are those with another AR event within 5 days before or after, and “sandwiched” ARs are those with AR events within 5 days both before and after (Fig. 1). In Fig. 2, the color of the dot represents the probability of an AR event being adjacent (top) or sandwiched (bottom), organized by AR intensity rank for every grid cell in California, where AR1 events are primarily beneficial and AR5 events are primarily hazardous. Dot size represents the number of ARs in that grid cell relative to the total number of statewide ARs of that rank from water year (WY) 1981 to 2021. Figure S1 repeats these results considering between-event intervals other than 5 days.

While the magnitude of the probabilities differs between the adjacent and the sandwiched cases, there are two trends visible in both scenarios. The first trend is that the probability of both adjacent and sandwiched events increases with increasing AR intensity, meaning that AR4 and AR5 events are more likely to have another AR before and/or after them. AR5 events are 1.7× more likely than AR1 events to be adjacent to at least one other AR and 2.6× more likely to be sandwiched between two ARs. The second trend is that the north coast, along the northwest edge of the state, tends to have higher probabilities of both adjacent and sandwiched ARs. There are more ARs in this region of the state across all intensity ranks, and compressing a larger number of events into the same time window will inevitably cause more compounding events.

**Fig. 3.**
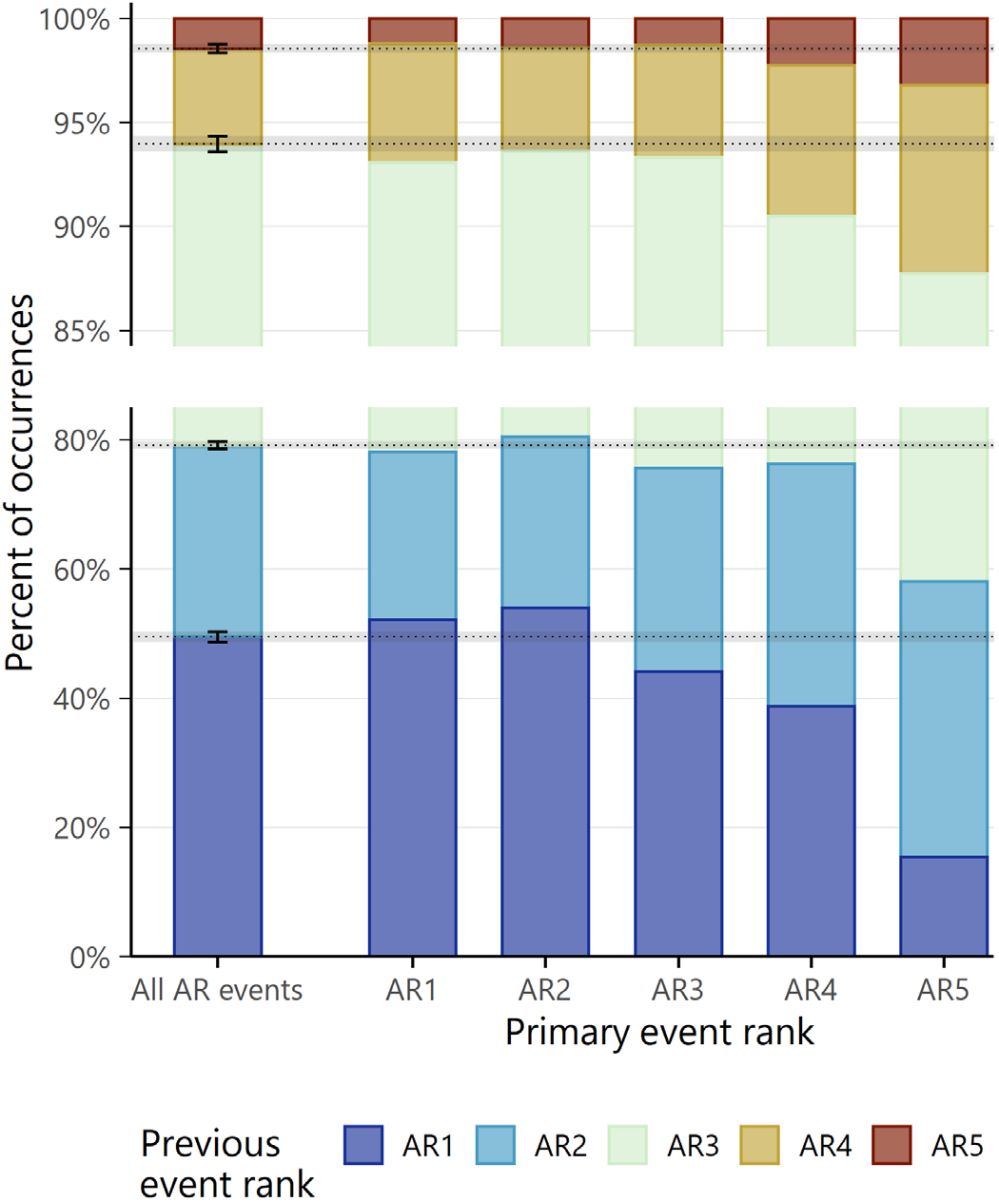
Relationship between primary event rank and previous event rank. This plot contains the subset of ARs in WY 1981 to 2021 that occur within 5 days of another AR (*N* = 10,616). The events are grouped by rank, and within each rank, we compare the observed distribution of previous event rank by primary event rank to the distribution of previous event rank derived from all events (dotted horizontal lines). Error bars and gray-shaded areas indicate bootstrapped 90% CIs. Note the discontinuous *Y* axis, which highlights trends in the rarer high AR ranks. We infer that ARs are more likely to cluster together with other ARs of similar intensities.

We also examine whether ARs of higher magnitudes tend to preferentially occur in close temporal proximity to other high-intensity ARs. The subset of AR events that occur within 5 days after another AR is grouped by intensity. These are referred to as the primary events, and the AR preceding each primary event is referred to as the previous event (Fig. 1). The overall distribution of previous event rank is shown on the left side of Fig. 3, labeled as “All AR Events,” with bootstrapped 90% CIs shown as black error bars and extended across the plot with gray shading. On the right, Fig. 3 shows the empirical distribution of previous event rank as a function of primary event rank.

If all AR events were equally likely to be preceded by events of any intensity, then the rank-specific bar heights in Fig. 3 would align with the dotted horizontal lines. Instead, there is a clear positive association between the ranks of the previous and primary events. AR1 and AR2 events are more likely to be preceded by an AR1 event. Conversely, in the top right corner of Fig. 3, AR4 and AR5 events are more likely to be preceded by other AR4 and AR5 events. Notably, AR5 events are three times less likely than the rank-agnostic expectation to be preceded by an AR1 but over two times more likely to be preceded by another AR5. Not only do high-intensity ARs tend to occur in close succession with other ARs generally, but they also tend to occur close to other high-intensity ARs. This finding has important implications for how we think about the impacts associated with high-rank AR events—If larger ARs are almost always part of a larger back-to-back series of events, then it might not be individual ARs causing impacts but rather the series itself.

### Sequences and probability of impact

We estimate the contribution of ARs and sequences to the probability of flood-related impact days, where an impact day is defined as a day where either the NFIP registers a claim or the NCEI Storm Events Database records a flood-related event. The NFIP claims contribute more impact days in densely populated coastal areas and around the city of Sacramento, where insurance take-up rates are high and there are large concentrations of policyholders who can submit claims. The NCEI database contributes more impact days along the eastern edge of the state, where the windward slopes of the Sierra Nevada mountains often experience severe winter storms. Approximately 5% of days from WY 1997 to 2021 caused an impact somewhere in the state, with the range in individual grid cells spanning from less than 1% of days to over 13%.

**Fig. 4.**
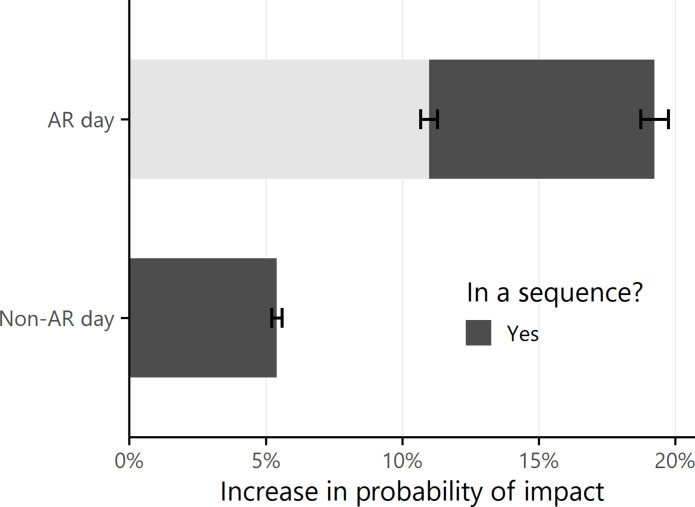
Effect of ARs and sequences on the probability of flood-related impact. The light gray bar shows the increase in probability of impact that can be attributed to ARs, independent of sequences, on all days from WY 1997 to 2021 (*N* = 1,506,615). The dark gray bars show the change in probability of impact that can be attributed to sequences on AR days (top) and non-AR days (bottom). Both bars show an increase in impact likelihood on days within sequences, with a larger increase on AR days than non-AR days. The error bars represent ±2 times the SE (approximately 90% CI).

The effects of ARs and sequences on flood impacts are estimated by fitting a linear probability model with fixed effects (Eq. 1 in Materials and Methods) at the daily scale. Days on which an AR occurs are referred to as AR days. Statewide, irrespective of sequences, AR days increase the probability of flood-related impact by 11.0% relative to non-AR days (Fig. 4), which agrees with the large body of evidence supporting the relationship between ARs, flooding, and flood loss. After the contribution of ARs is accounted for, sequences increase probability of impact by an additional 8.3% on AR days, relative to the expected probability on AR days outside of a sequence. This means that an AR day within a sequence is 11.0 + 8.3 = 19.3% more likely to cause flood-related impact than a non-AR day outside of a sequence (Fig. 4). The probability of impact on non-AR days within a sequence is 5.4% higher than that on non-AR days outside of a sequence. The persistent effect of sequences on impact likelihood even during non-AR days may be related to the delay that can occur between AR events and their hydrologic impacts (*35*). Figure 4 illustrates these coefficient values and their associated 90% CIs.

### Sequences and magnitude of loss

**Fig. 5.**
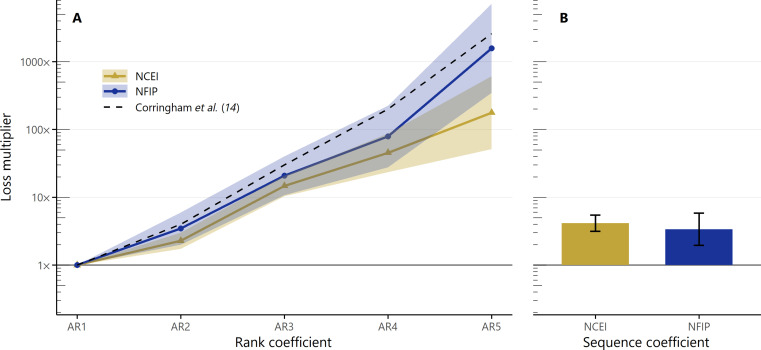
Effect of AR rank and sequences on loss. (**A**) Fitted coefficient and 90% CI for the multiplicative change in loss (loss multiplier) expected from an AR2 to AR5 relative to an AR1, calculated from NCEI database (gold triangles) and NFIP claims data (dark blue circles) (*N* = 23,267). The dashed line shows the loss estimates by rank from Corringham *et al.* (*14*), reformatted as loss multipliers, i.e., the values shown for AR2 through AR5 are the median loss estimates for those ranks divided by the median loss estimate for AR1. The loss multipliers increase exponentially with increasing magnitude, and the NFIP claims data consistently estimate slightly higher coefficients than the NCEI database. (**B**) Fitted coefficient (bar) and 90% CI (error bar) for the multiplicative change in loss expected for an AR of any magnitude falling within a sequence relative to an AR outside of a sequence. The loss multiplier calculated from the NCEI database is larger and has a narrower CI than the one calculated from the NFIP claims data.

We next estimate the magnitude of loss by fitting linear regressions with fixed effects to loss data from both the NCEI Storm Events Database and NFIP insurance claims (Eq. 2). Figure 5A compares the fitted coefficients for AR2 to AR5 events as calculated using the storm-total loss estimates from the NCEI database (gold) and payouts from NFIP claims (dark blue). These coefficients represent the multiplicative increase in loss relative to what would be experienced from an AR1 event. The shaded area represents a 90% CI around each coefficient estimate.

Losses increase exponentially as a function of AR magnitude across both datasets. There is good agreement between the results from our analysis of the NFIP claims data and the results from Corringham *et al.* (*14*), shown as a black dashed line in Fig. 5, which were also determined on the basis of NFIP claims. We find that losses from AR5 events are approximately 1600× larger than those from AR1 events (90% CI: 350× to 7100×), compared to a loss multiplier of 2600× in Corringham *et al.* (*14*). The regression coefficients calculated using the NCEI database are universally lower: for AR2, AR3, and AR4 events, loss coefficients are 30 to 40% smaller than those from the NFIP claims data, and for AR5 events, the difference is almost a full order of magnitude. This could be due to methodological differences between the two datasets; because loss values in the NCEI database are usually based on preliminary loss estimates, they are more likely to be biased low, especially for larger events where it takes more time for complete losses to be tabulated (*41*). Overall, though, these coefficients match our expectations about the effect of AR intensity on the severity of flood-related losses in California.

Figure 5B shows the fitted coefficients for the binary variable indicating whether an AR falls within a sequence, relative to an AR that does not fall in a sequence, with the 90% CIs shown as error bars. Expected losses for ARs in sequences are 4.2× larger based on the NCEI database (90% CI: 3.2× to 5.5×) and 3.4× larger based on NFIP claims data (90% CI: 1.9× to 5.8×) than losses for ARs outside of sequences. These values are roughly the same as the losses expected due to increasing from AR1 to AR2 or from AR3 to AR4. Sequences thus cause a sizeable and statistically significant increase of overall loss in both datasets. Note also that these are multiplicative factors applied on top of the rank-specific coefficients presented in Fig. 5A; the effect of being in a sequence, therefore, increases with increasing event magnitude and makes the largest difference for the loss totals of the most damaging ARs.

The magnitude of a given AR and whether that AR falls within a sequence are correlated, as shown in the analysis of temporal compounding and AR rank in Fig. 2 and 3. Table 1 reports the number of ARs with nonzero loss in either dataset by both rank and sequence status. While only a portion of the low-intensity ARs that cause nonzero loss fall within sequences, almost all high-intensity ARs that cause nonzero loss do. This is a notable feature on its own, as it indicates the tight coupling between large damaging storms and sequences. It also creates challenges in statistical analysis, as care is required to separate the role of sequences from the role of AR intensity. We address this challenge by using one sequence coefficient for all ARs rather than attempting to estimate rank-specific effects. While the effects of sequences cannot be fully untangled from AR magnitude, the results presented in this section support the qualitative expectation that sequences are important drivers of loss.

**Table 1.** Relationship between sequence, rank, and loss. Data include ARs associated with either a nonzero NCEI storm event or a nonzero NFIP claim from WY 1997 to 2021 (*N* = 2113).

AR rank	Within sequences	Outside sequences
AR1	341	446
AR2	451	168
AR3	493	36
AR4	129	1
AR5	48	0

### Effect of previous event characteristics on loss

**Fig. 6.**
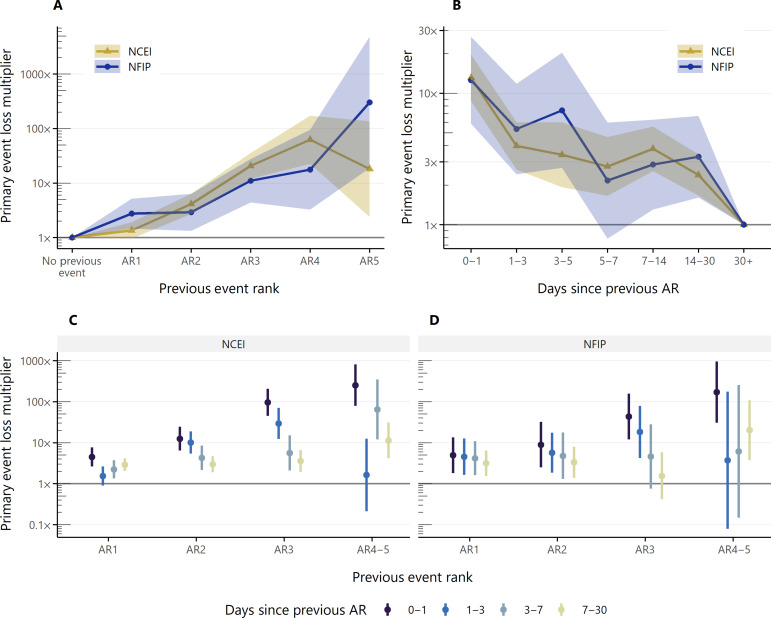
Effect of previous AR characteristics on loss. (**A**) Effect of the AR intensity rank of the previous event on primary event losses (*N* = 23,267). The NCEI database (gold triangles) and NFIP claims data (dark blue circles) both estimate increasing loss multipliers with increasing previous event magnitude. (**B**) Effect of the time since previous event (between-event interval) on primary event losses. The NCEI database (gold triangles) and NFIP claims data (dark blue circles) both estimate a decreasing effect on primary losses with increasing between-event interval. (**C** and **D**) Interacting effect of previous event intensity and time since the previous event, where (C) shows results from the NCEI database and (D) shows results from the NFIP claims data. Both plots show increasing sensitivity of primary event losses at higher intensity ranks and shorter between-event intervals.

To dive deeper into the impacts of temporal compounding, we look at the relationship between an AR event (the primary event) and the one immediately preceding it (the previous event). Figure 6A first focuses on how the intensity of the previous event affects the loss associated with the primary event. We consider the subset of ARs that occur within 5 days of another AR event (the same subset used to create Fig. 2 and 3. and calculate loss coefficients for the primary AR as a function of the intensity rank of the previous AR. Figure 6A reveals that the larger the magnitude of the previous event, the stronger its impact on the primary event’s loss total. Coefficients calculated from both the NCEI database and the NFIP claims data largely agree on this increasing trend, except for the downward dip for AR5 events seen in the NCEI database coefficients. To contextualize the magnitude of these coefficients, we compare the effect of the previous event being an AR5 to the effect of the primary event being an AR5. We start with a comparison based on the NFIP claims data. We saw in Fig. 5A that an AR5 event has 1600× the loss of an AR1 event. In Fig. 6A, an AR preceded by an AR5 event has 300× the loss of an AR without a preceding event. Therefore, the previous-event coefficient is 19% of the primary-event coefficient. Repeating this calculation for the NCEI database shows that the previous coefficient is 10% of the primary coefficient. These are sizeable potential contributions that help to explain some of the variance that appears in an event-specific analysis of losses.

Figure 6B considers the effect of the time between the previous and primary events (between-event interval) on the loss associated with the primary event. We found that the between-event interval was approximately lognormally distributed and chose thresholds that divided the data into similarly sized bins, separated at 1, 3, 7, and 30 days. The trend in Fig. 6B decreases as the inter-event window between the primary and previous AR increases. The regression coefficients shown here are calculated relative to the expected loss from an AR with a preceding between-event interval of 30 days or more, which represent 37% of all AR events. Overall, while coefficients from the NFIP claims data are more variable and have larger CIs than those from the NCEI database, both datasets support that longer intervals between the previous event and the primary one lower the previous event’s effect on primary event losses.

In Fig. 6 (C and D), we combine previous event intensity and between-event interval into a single regression to explore interaction effects between these two contrasting influences. All coefficients are relative to losses from ARs with no previous event within 30 days. Including interactions substantially increases the number of regression coefficients that need to be estimated, and the model becomes poorly constrained at higher magnitudes, where there are fewer records in the dataset. Therefore, we combine previous AR4 and AR5 events into one group labeled “AR4-5.” We found that these modifications were able to show the trends that emerged from interaction while reducing noise and constraining CIs around the coefficient estimates.

Moving from lower to higher magnitudes, the within-group coefficient spread increases in both Fig. 6C (NCEI) and Fig. 6D (NFIP) and develops a downward slope as a function of between-event interval. The dark blue lines representing a 0- to 1-day interval trend steadily upward, while the yellow lines representing a 7- to 30-day interval stay relatively flat. The loss associated with the primary event is therefore more sensitive to previous event rank when the between-event interval is smaller. At longer intervals, there is still a positive contribution from the previous event, but the contribution is less dependent on the rank of that event. The largest coefficient across both datasets is, expectedly, the effect of AR4-5 events within the last 0 to 1 days: 250× for the NCEI database and 170× for the NFIP claims data. These represent the highest-intensity events at the shortest time interval. More generally, AR3 to AR5 events that occur within about a week of the primary event can increase expected losses by an order of magnitude or more. The smallest coefficients, while not significantly different than zero, are all positive, consistent with Fig. 6 (A and B). Overall, losses associated with ARs occurring within 30 days of a preceding event will almost always be higher than that of ARs without a preceding event, and the magnitude of the loss multiplier is jointly controlled by the preceding event’s intensity and recency.

### Effect of sequence position on loss

**Fig. 7.**
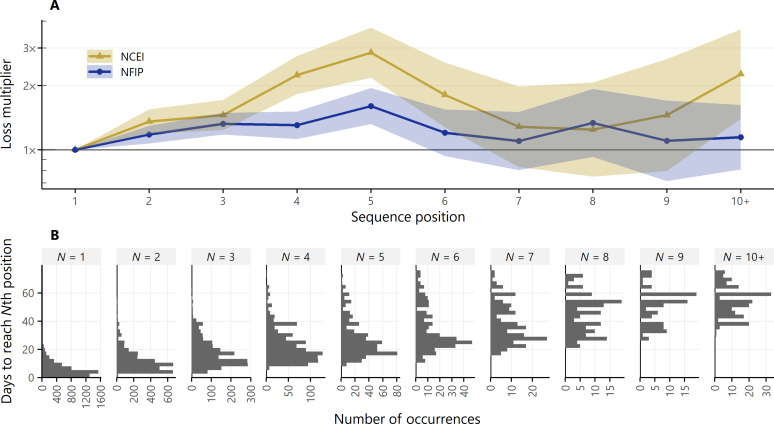
Effect of AR sequence position on loss. (**A**) Fixed effects regression coefficients for the NCEI database (gold triangles) and the NFIP claims data (dark blue circles), estimating the additional loss expected from the second or later AR in a sequence relative to the first AR in a sequence. (**B**) Histograms of the number of days taken to reach the *N*th sequence position, from *N* = 1 to *N* = 10+.

Our final analysis looks only at the subset of ARs that occur within sequences (*N* = 12,745; 55% of the full catalog) and calculates the effect of sequence position on losses. Starting from the first AR in a sequence and moving toward subsequent ARs, regression coefficients for both the NCEI database and the NFIP claims data increase almost monotonically, as shown in Fig. 7A. The second AR in the sequence has higher expected losses than the first, the third has higher expected losses than the second, etc. This pattern continues until the fifth sequence position in both datasets, where the loss multiplier peaks at 2.8× (NCEI) and 1.6× (NFIP). We recall from the analysis of loss magnitude in Fig. 5B that sequences increase losses by roughly three to four times. Because this regression is only performed on ARs within sequences, the loss multipliers here are applied on top of that value, so these have the potential to be very large losses in absolute terms. For ARs beyond the fifth sequence position, there are fewer events to constrain the coefficient estimates, so the CIs start to widen and the coefficients lose statistical significance at the sixth (NFIP) and seventh (NCEI) positions. The coefficient estimates remain positive for every position, though, reinforcing the importance of temporal compounding and watershed memory in loss estimation.

We relate sequence position to sequence duration in Fig. 7B. These plots show the distribution of the difference between the start date of the sequence and the start date of the AR in the *N*th sequence position. For example, the fifth AR occurs a median of 25 days into the sequence, implying that the cumulative effect of multiple past ARs can influence the primary event for weeks to months. While the preceding event affects losses for the primary AR, an AR’s impacts can continue to be influenced by more than just the most recent previous AR. Looking at position allows us to take a more long-term view and account for the effects of multiple past events at once.

## DISCUSSION

This paper offers several insights into the relationship between temporally compounding ARs and economic loss. First, high-intensity ARs are more likely to occur in close temporal proximity to other ARs. Statewide, AR5 events are 1.7× more likely than AR1 events to have another AR within 5 days before or after (adjacent case) and 2.6× more likely to have another AR within 5 days before and after (sandwiched case). High-rank ARs also tend to cluster together with other high-rank ARs. Practically, this means that the events with the most negative hydrologic and economic consequences are likely to occur more than one at a time and to do so in such rapid succession that there is no time for the hydrologic environment to recover.

Second, if an AR falls within a sequence (indicating higher potential for temporal compounding), it increases both the likelihood and magnitude of economic impacts for that AR. We use a series of fixed-effects regressions to control for spatial and temporal differences in losses across California from WY 1997 to 2021 and isolate the contribution of sequences to impact days and loss totals. Being in a sequence increases the likelihood of impact by 5.4% on non-AR days and 8.3% on AR days and more than triples the expected loss associated with a given AR event. To generate these results, we assessed losses independently across two separate datasets. The two datasets differ in their spatiotemporal scales and data collection protocols, but despite these differences, the regression results calculated separately for each dataset universally agree on the direction and magnitude of the effect of sequences, converging on an increase in damages of roughly three to four times. This convergence increases the robustness and reliability of our results.

Last, the characteristics of the AR event(s) preceding the one under consideration materially affect expected losses. We examined how the magnitude of the previous event and the time since the previous event affected the loss total from the primary event. More intense previous ARs and shorter between-event intervals lead to larger increases in primary event losses, and the effect of the preceding AR fades but is still positive for between-event intervals up to a month or more. Total losses for a given AR are additionally influenced by not just the event immediately preceding it but also its overall placement relative to other ARs in a sequence. Losses increase as a function of sequence position up to the fifth AR in a sequence; on top of the 3× to 4× loss multiplier that is applied due to being within a sequence, being the fifth AR in sequence increases losses by an extra 1.5× to 3× relative to the first AR in the sequence. Incorporating knowledge about prior events can reduce uncertainty when predicting the impacts of the next event, which is critically important for water management and emergency preparation.

We have presented multiple lines of evidence to illustrate the substantial role temporal compounding plays in modulating AR impact and loss. Together, these findings highlight the importance of considering the larger context around individual AR events to understand their loss potential, especially as temporal compounding increases in frequency and severity in a warming climate.

## MATERIALS AND METHODS

### Hazard data

IVT was calculated from the Modern-Era Retrospective Analysis for Research and Applications, version 2 (MERRA-2) (*42*). MERRA-2 covers the globe at a horizontal grid resolution of 0. 5^∘^ × 0.625^∘^ (~50 km by 50 km) and reports data from WY 1981 to 2021 at a 3-hour time step. We used the Rutz *et al.* (*38*) detection algorithm to identify ARs independently in all MERRA-2 grid cells intersecting with California and calculated the Ralph *et al.* (*15*) ranking for each identified event. AR objects were not tracked across cells. “Weak” ARs with IVT < 500 kg m^−1^ s^−1^ and duration < 24 hours were removed from the analysis. Sensitivity analyses exploring the effect of alternative AR detection algorithms on key results are included in figs. S2 and S3 of the Supplementary Materials.

AR sequences were identified on the basis of the method outlined by Bowers (*35*) and are described briefly here. In each MERRA-2 grid cell, we calculate the 5-day moving average IVT and identify the intervals when the moving average exceeds the 30-year median IVT for that cell. The maximum moving average IVT is determined within each of these intervals, and intervals where the maximum exceeds 250 kg m^−1^ s^−1^ are determined to be sequences. The choice of a 5-day window is both consistent with prior work on temporally compounding ARs (*36*) and focuses on compounding at the scale of days to weeks, where runoff levels and, to a lesser extent, soil moisture modulate the relationship between hazard and impact. It also limits the influence of individual large ARs, which, by definition, can only affect the moving average for 2.5 days before and after the event. Bowers (*35*) provide an in-depth discussion of the sensitivity of the sequences metric to different values of these parameters. The benefits of using a moving average–based approach, rather than a count-based approach as proposed by others (*36*, *43*), include a measure of intensity other than duration, the ability to capture additional hydrologic consequences beyond the end of AR conditions, and results that are independent of a specific AR detection algorithm.

### Impacts data

To balance the limitations inherent to all flood loss estimates (*44*–*46*), we used two datasets of economic loss. The first is the NCEI Storm Events Database (*39*), which is a catalog of events from the National Weather Service (NWS) Storm Data Publication that includes post-event estimates of total losses. We retained NWS events in the following hazard categories: blizzard, flash flood, flood, heavy rain, heavy snow, winter storm, and winter weather. While the NCEI Storm Events Database goes back to 1950, the hazard categories used in this analysis were not included until 1996. Therefore, we start our analysis of impacts and loss in WY 1997, the first WY with complete data. The second is the record of NFIP flood insurance claims retrieved from the Federal Emergency Management Agency’s (FEMA) OpenFEMA data platform (*40*). NFIP claims are available starting in 1978 but are limited to insured properties, which are largely standalone residential buildings. Claims are recorded at the property level rather than the county level and are tagged to the date that the loss occurred, even if the claim itself is submitted later.

NFIP claims are arguably the most common choice for flood loss assessment in the United States (*47*–*50*). They have been used extensively in AR loss estimation, most notably by Corringham *et al.* (*14*) in their estimate of loss by AR event intensity. The NCEI database is used less frequently and is often limited to the assessment of impacts (i.e., the presence of a flood); for example, Young *et al.* (*51*) compared ARs in California against days with NCEI flood events, and Dougherty and Rasmussen (*52*) matched patterns of extreme rainfall with NCEI flood events in different climatological regions of the US. There are some studies that use the NCEI dataset or other closely related datasets also derived from the NWS Storm Data Publication to assess magnitude of loss (*53*, *54*) but none related specifically to ARs. The two datasets differ considerably in structure because of the differences in agency missions and data collection procedures. While NFIP claims are never explicitly tied to a specific meteorological event, NCEI losses always are; NCEI losses are also reported by county/NWS weather forecast zone rather than by day and point location. Therefore, the NCEI Storm Events Database may not capture events with nonzero losses that are not deemed meteorologically noteworthy, but it does include storms that are theoretically large enough to cause losses but do not result in any insurance claims.

Both datasets were subsetted to California and adjusted to 2022 dollars and then spatially and temporally aggregated to match the MERRA-2 grid at a daily scale. In the NCEI database, events can be reported in the database either by county or by NWS weather forecast zone. While California county boundaries have not changed since 1996, NWS forecast zones have, and a best-effort attempt was made to match events with the zone map in effect at that time. If a county or a zone crossed multiple MERRA-2 grid cells, then the loss was divided spatially based on a weighted average. Losses from multiday NCEI events were also equally divided temporally over all days in that event to create a daily dataset of NCEI losses. We kept two calculated variables: one indicating whether there was an NCEI event recorded at that place and time (impacts) and one measuring the spatially and temporally disaggregated total loss (losses). For the NFIP, we calculated both the total number of claims including denied claims or claims with zero payout (impacts) and the total value of claims paid out (losses) for each day and each grid cell.

### Aggregation

We created two products for every MERRA-2 grid cell in California to perform the analysis presented in the results. The first was a daily time series indicating the days labeled as AR days/sequence days, which was used to estimate the effect of sequences on probability of damage. A day was considered an AR day and/or a sequence day if at least two 3-hour periods in the 24-hour window were labeled as an AR or a sequence, respectively. The daily time series dataset was matched with gridded daily impact data from the NFIP claims dataset and the NCEI Storm Events Database to estimate the effect of sequences on the probability of impact days. The second was an AR event catalog, which was used to quantify the contribution of sequences to loss magnitude. To create the AR event catalog, we assigned an ID number to each AR event, calculated its intensity rank based on maximum IVT and duration, recorded whether it was in a sequence, and summed each loss variable to measure AR-total NFIP and NCEI loss. Losses that occur on days not associated with ARs were discarded from further analysis. We also paired each AR with the one preceding it to create two new variables: the interval, in days, between the end of the preceding AR and start of the current AR, and the intensity rank of the preceding AR. The catalog was matched with gridded daily loss data from the NFIP claims dataset, and the NCEI Storm Events Database and values were summed to generate AR-total losses.

We modified the AR event catalog to address the severe imbalance between zero-loss events and nonzero-loss events. The proportion of ARs with nonzero NCEI loss is 7.8%, and the proportion with nonzero NFIP loss is 2.1%, which made it difficult to calculate stable regression coefficients and report results from interaction effects. We undersampled the data and randomly removed records from the majority class (zero loss) to reach a class balance of 33%. Random undersampling has a long track record of successful use in data mining and machine learning with imbalanced data, and it reliably achieves similar levels of prediction accuracy when compared to other class rebalancing methods (*55*, *56*). We repeated the undersampling process 1000 times for each event-based regression to bootstrap coefficient estimates and CIs.

### Regressions

All regressions presented in this paper are fixed-effects regression models with spatial and temporal variability removed. Regressions with fixed effects have been used frequently to understand the relationship between environmental phenomena and societal impacts (*53*, *57*–*59*). The spatial fixed effect (γ*_x_*), removed for each MERRA-2 grid cell, represents cell-specific deviations from the statewide mean and accounts for spatial differences due to hazard (e.g., climatological norms), exposure (e.g., population density and development patterns), and vulnerability (e.g., flood risk awareness and mitigation investment). The temporal fixed effect (δ*_y_*) represents statewide annual trends and accounts for differences due to both internal changes in the datasets (e.g., updates to data reporting standards) and external factors (e.g., population growth or decline). Annual trends are removed by WY, which starts on 1 October and goes through September of the following calendar year.

The regression for determining impact day likelihood is a linear probability model, which is the form recommended by Timoneda (*60*) for fixed-effects models with highly imbalanced binary dependent variables. The equation is as follows

$$p_{\mathrm{xt}}=\beta_{1}AR_{\mathrm{xt}}+\beta_{2}\left(AR_{\mathrm{xt}}*\text{SE}Q_{\mathrm{xt}}\right)+\beta_{3}\left({\bar{\text{AR}}}_{xt}*\text{SE}Q_{\mathrm{xt}}\right)+\gamma_{x}+\delta_{y}+\epsilon_{\mathrm{xt}}$$ (1)

where *p_xt_* is the probability of impact at grid cell *x* on day *t*; *AR_xt_* is the binary indicator of an AR at grid cell *x* on day *t*; β_1_ is the loss multiplier for an AR day, relative to a non-AR day; *SEQ_xt_* is the binary indicator of a sequence at grid cell *x* on day *t*; β_2_ is the loss multiplier for sequenced AR days, relative to nonsequenced AR days; ${\bar{\text{AR}}}_{\mathrm{xt}}$ is the inverse of the binary indicator *AR_xt_*, representing a non-AR day; β_3_ is the loss multiplier for sequenced non-AR days, relative to nonsequenced non-AR days; γ*_x_* is the spatial fixed effect removing variation across grid cells; δ*_y_* is the temporal fixed effect removing annual variation by WY; and ϵ*_xi_* is the residual error term.

Equations 2 through 6 are all ordinary least squares linear regressions rather than linear probability models, meaning that they predict loss magnitude instead of probability, and the coefficients are calculated on a location-event basis rather than a location-day basis. The fixed-effects regression used to estimate the effect of sequences on loss is a function of AR intensity rank and a binary variable indicating whether the AR is in a sequence. The outcome variable is the logarithm of NCEI/NFIP losses. Structuring the equation to predict a log value offers two benefits: It transforms the right-skewed loss data to be approximately normal, which satisfies the assumptions of linear regression, and it generates regression coefficients that represent a relative change in loss rather than an absolute one. With relative coefficients, we can directly compare the results from the NCEI loss data and the NFIP loss data without having to explicitly account for all of the factors driving difference in absolute magnitude between the two datasets. The regression equation used to predict loss magnitude is as follows

$$\text{log}\left(L_{\mathrm{xi}}\right)=\sum_{r=2}^{5}‍\alpha_{r}\;\left(AR_{\mathrm{rxi}}\right)+\beta \;\left(\text{SE}Q_{\mathrm{xi}}\right)+\gamma_{x}+\delta_{y}+\epsilon_{\mathrm{xi}}$$ (2)

where *L_xi_* is the NCEI/NFIP loss in 2022 dollars at grid cell *x* for event *i*; *AR_xi_* is the binary indicator of an AR of rank *r* at grid cell *x* for event *i*; α*_c_* is the loss multiplier for ARs of rank *r*, relative to AR1; *SEQ_xi_* is the binary indicator of a sequence at grid cell *x* for event *i*; and β is the loss multiplier for ARs in sequences, relative to ARs not in sequences. Coefficients are calculated separately for NCEI and NFIP data.

The regression equations for estimating the impact of previous event intensity, between-event interval, and the interactions between the two are as follows

$$\text{log}\left(L_{\mathrm{xi}}\right)=\sum_{r=2}^{5}‍\alpha_{r}\;\left(AR_{\mathrm{rxi}}\right)+\sum_{r=1}^{5}‍\beta_{\mathrm{rd}}\;\left(\text{PRE}V_{\text{rdxi}}\right)+\gamma_{x}+\delta_{y}+\epsilon_{\mathrm{xi}}$$ (3)

$$\text{log}\left(L_{\mathrm{xi}}\right)=\sum_{r=2}^{5}‍\alpha_{r}\;\left(AR_{\mathrm{rxi}}\right)+\sum_{d\in D}‍\beta_{d}\;\left(\text{IN}T_{\text{dxi}}\right)+\gamma_{x}+\delta_{y}+\epsilon_{\mathrm{xi}}$$ (4)

$$\text{log}\left(L_{\mathrm{xi}}\right)=\sum_{r=2}^{5}‍\alpha_{r}\;\left(AR_{\mathrm{rxi}}\right)+\sum_{r=1}^{5}‍\sum_{d\in D}‍\beta_{\mathrm{rd}}\;\left(\text{PRE}V_{\text{rdxi}}*\text{IN}T_{\mathrm{dxi}}\right)+\gamma_{x}+\delta_{y}+\epsilon_{\mathrm{xi}}$$ (5)

Equation 3 measures the effect of previous event rank on primary event losses, Eq. 4 measures the effect of time since previous event on primary event losses, and Eq. 5 measures the interacting effects of previous event rank and time since previous event on primary event losses. *PREV_rdxi_* is the binary indicator of an AR of rank *r* preceding the primary event within some between-event interval *d* at grid cell *x* for event *i*; *INT_dxi_* is the binary indicator of falling within interval *d* at grid cell *x* for event *i*; β*_rd_* is the loss multiplier for the previous event being an AR of rank *r*, relative to the expected loss if no AR had occurred within interval *d*; and *D* is the set of intervals considered for *d*.

The regression equation for estimating the impact of sequence position on loss is as follows

$$\text{log}\left(L_{\mathrm{xi}}\right)=\sum_{r=2}^{5}‍\alpha_{r}\;\left(AR_{\mathrm{rxi}}\right)+\sum_{n=2}^{10}‍\beta_{n}\left(\text{PO}S_{\mathrm{nxi}}\right)+\gamma_{x}+\delta_{y}+\epsilon_{\mathrm{xi}}$$ (6)

where *POS_nxi_* is the binary indicator for the AR at the *n*th position in a sequence at grid cell *x* for event *i*; and β*_n_* is the loss multiplier for being the *n*th AR in a sequence, relative to the average loss experienced during the first AR in a sequence. *n* = 10 represents the catchall category of ARs in the 10th position or later (1.5% of all ARs falling within sequences).

## Supplementary Materials

### This PDF file includes:

Figs. S1 to S3 References
