# Supplementary material for: Temporal compounding increases economic impacts of atmospheric rivers in California
Source: Sci Adv. 2024 Jan 19;10(3):eadi7905. doi: 10.1126/sciadv.adi7905 (PMC10798553; doi:10.1126/sciadv.adi7905)
Supplement: Supplementary file 1 — Figs. S1 to S3 References [file sciadv.adi7905_sm.pdf]

Science Advances
AAAS

Supplementary Materials for

Temporal compounding increases economic impacts of atmospheric rivers in California

Corinne Bowers *et al.*

Corresponding author: Corinne Bowers, cbowers@usgs.gov

Sci. Adv. **10**, eadi7905 (2024)
DOI: 10.1126/sciadv.adi7905

This PDF file includes:

Figs. S1 to S3
References

Fig. S1.

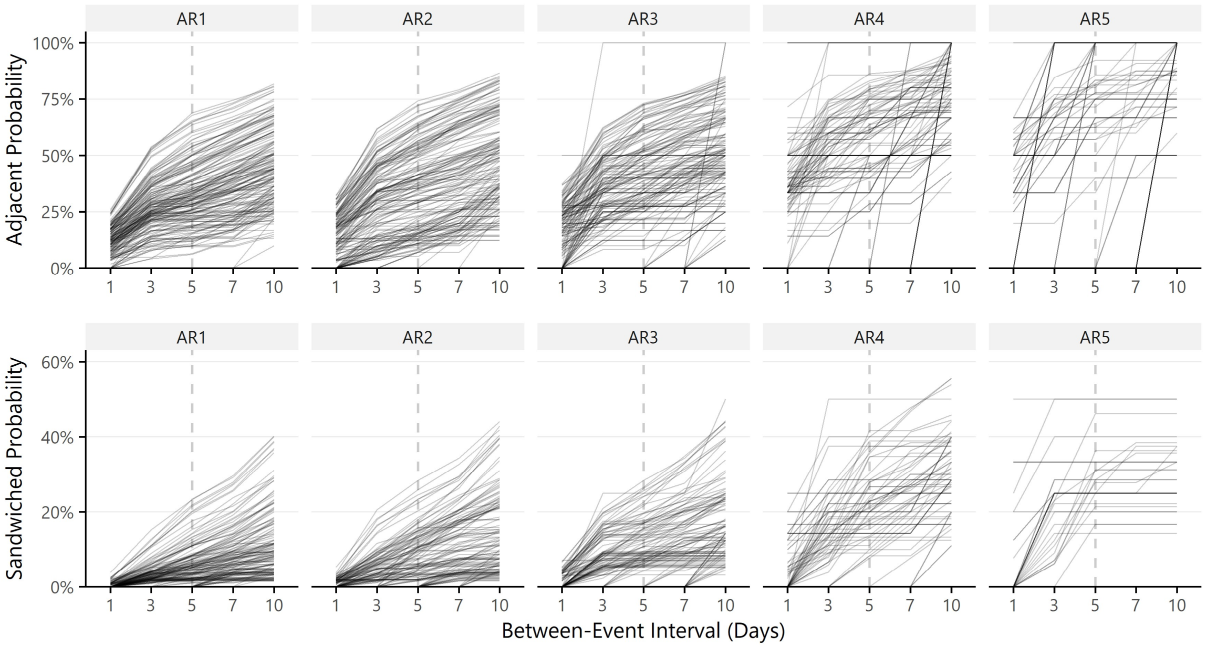

Figure S1: Effect of between-event interval on probability of adjacent ARs and probability of sandwiched ARs. Each line represents one of the MERRA-2 grid cells shown spatially in Figure 2, and the dashed vertical line indicates the five-day interval reported in Figure 2. The probability of adjacent ARs (top row) and sandwiched ARs (bottom row) increases with increasing between-event interval. The rate of increase appears to be about the same across different AR intensities.

Fig. S2.

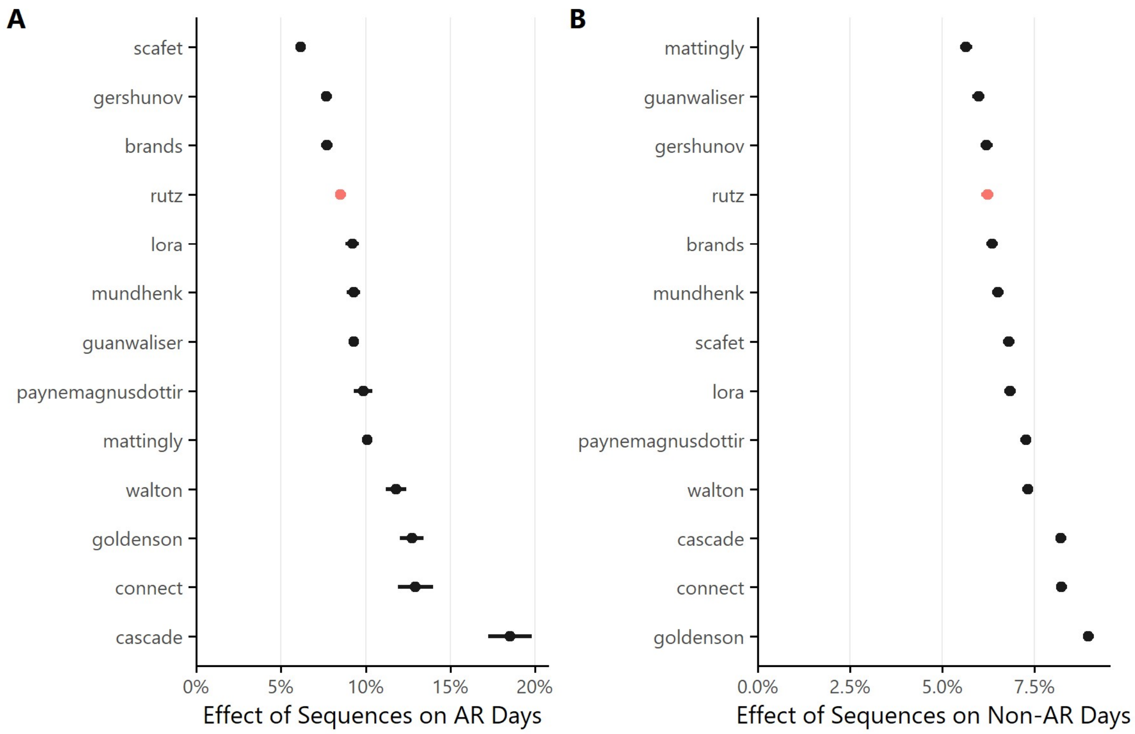

Figure S2: Sensitivity of the effect of sequences on probability of flood-related impact to different AR detection algorithms. Bars represent the mean and 90% confidence interval of the estimated increase in probability of impact on (**A**) AR days within sequences relative to AR days outside of sequences (β_2 in Eq. 1) and (**B**) non-AR days within sequences relative to non-AR days outside of sequences (β_3 in Eq. 1). The *rutz* values, which correspond to the Rutz et al. *(38)* results shown in Figure 4 of the main text, are shown in pink. These values are compared against results from twelve additional algorithms archived as part of the AR Tracking Method Intercomparison Project (ARTMIP) Tier 1 *(62)*. The names of the algorithms and their ARTMIP identification numbers *(63)* are as follows: *brands* [A24], *cascade* [A25], *connect* [A12], *gershunov* [A1], *goldenson* [A2], *guanwaliser* [A3], *lora* [A7], *mattingly* [A39], *mundhenk* [A19], *paynemagnusdottir* [A8], *scafet* [A38], and *walton* [A22]. Algorithms are ordered by increasing coefficient size in each plot. Note that the ARTMIP Tier 1 has only archived AR detection results through 2016 while the manuscript shows results through 2021, so the *rutz* coefficients calculated as part of this sensitivity analysis will not exactly match the coefficients shown in the manuscript.

Fig. S3.

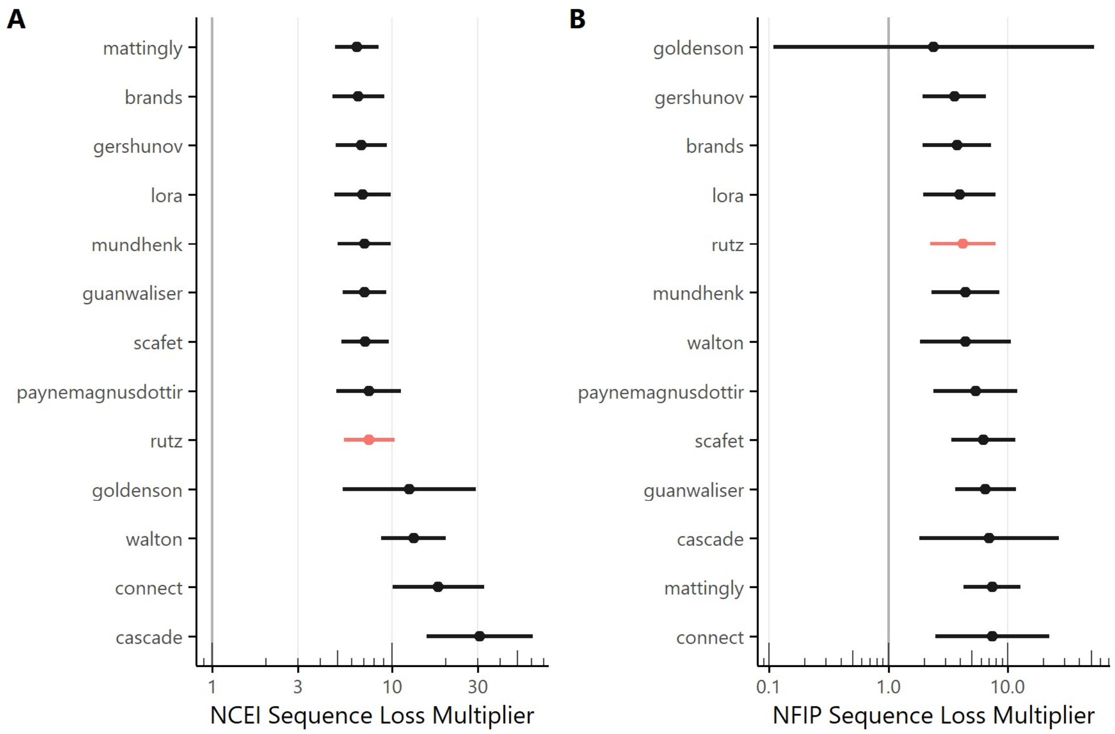

Figure S3: Sensitivity of the effect of sequences on loss to different AR detection algorithms. Bars represent the mean and 90% confidence interval of the estimated multiplicative change in loss expected for an AR within a sequence relative to an AR outside of a sequence (β in Eq. 2), calculated using **(A)** the NCEI database and **(B)** NFIP claims data. The *rutz* values, which correspond to the Rutz et al. *(38)* results shown in Figure 4 of the main text, are shown in pink. These values are compared against results from twelve additional algorithms archived as part of the AR Tracking Method Intercomparison Project (ARTMIP) Tier 1 *(62)*. The names of the algorithms and their ARTMIP identification numbers *(63)* are as follows: *brands* [A24], *cascade* [A25], *connect* [A12], *gershunov* [A1], *goldenson* [A2], *guanwaliser* [A3], *lora* [A7], *mattingly* [A39], *mundhenk* [A19], *paynemagnusdottir* [A8], *scafet* [A38], and *walton* [A22]. Algorithms are ordered by increasing coefficient size in each plot. Note that the ARTMIP Tier 1 has only archived AR detection results through 2016 while the manuscript shows results through 2021, so the *rutz* coefficients calculated as part of this sensitivity analysis will not exactly match the coefficients shown in the manuscript.

REFERENCES AND NOTES

1. M. A. Lamjiri, M. D. Dettinger, F. M. Ralph, B. Guan, Hourly storm characteristics along the U.S. West Coast: Role of atmospheric rivers in extreme precipitation. *Geophys. Res. Lett.* **44**, 7020–7028 (2017).

2. B. Liu, X. Ma, Z. Duan, J. Jiang, M. Reichstein, M. Jung, Impact of temporal precipitation variability on ecosystem productivity. *WIREs Water* **7**, 1 (2020).

3. X. Chen, L. Ruby Leung, Y. Gao, Y. Liu, M. Wigmosta, M. Richmond,Predictability of extreme precipitation in western U.S. watersheds based on atmospheric river occurrence, intensity, and duration. Geophysical research letters **45**, 693–11 (2018).

4. M. D. Dettinger, Climate change, atmospheric rivers, and floods in California—A multimodel analysis of storm frequency and magnitude changes1 *JAWRA.* **47**, 514 (2011), 523.

5. L. R. Leung, Y. Qian, Atmospheric rivers induced heavy precipitation and flooding in the western U.S. simulated by the WRF regional climate model *Geophys. Res. Lett.* **36**, 1 (2009).

6. C. P. Konrad, M. D. Dettinger, Flood runoff in relation to water vapor transport by atmospheric rivers over the western United States 1949–2015 *Geophys. Res. Lett.* **44**, 11,456–11,462(2017).

7. X. Chen, L. R. Leung, M. Wigmosta, M. Richmond, Impact of atmospheric rivers on surface hydrological processes in western U.S. watersheds *J. Geophys. Res. Atmos.* **124**, 8896–8916 (2019).

8. C. M. Albano, M. D. Dettinger, A. A. Harpold, Patterns and drivers of atmospheric river precipitation and hydrologic impacts across the western united states *J. Hydrometeorol.* **21**, 143 (2020).

9. D. L. Swain, D. E. Horton, D. Singh, N. S. Diffenbaugh, Trends in atmospheric patterns conducive to seasonal precipitation and temperature extremes in California *Sci. Adv.* **2**, e1501344 (2016).

10. A. Gershunov, T. Shulgina, R. E. S. Clemesha, K. Guirguis, D. W. Pierce, M. D. Dettinger, D. A. Lavers, D. R. Cayan, S. D. Polade, J. Kalansky, F. M. Ralph, Precipitation regime change in western North America: The role of atmospheric rivers *Sci. Rep.* **9**, 9944 (2019).

11. G. G. Persad, D. L. Swain, C. Kouba, J. P. Ortiz-Partida, Inter-model agreement on projected shifts in California hydroclimate characteristics critical to water management *Clim. Change* **162**, 1493–1513 (2020).

12. X. Huang, D. L. Swain, A. D. Hall, Future precipitation increase from very high resolution ensemble downscaling of extreme atmospheric river storms in California *Sci. Adv.* **6**, eaba1323 (2020).

13. A. M. Rhoades, A. D. Jones, A. Srivastava, H. Huang, T. A. O'Brien, C. M. Patricola, P. A. Ullrich, M. Wehner, Y. Zhou, The shifting scales of western U.S. landfalling atmospheric rivers under climate change *Geophys. Res. Lett.* **47**, 1 (2020).

14. T. W. Corringham, F. M. Ralph, A. Gershunov, D. R. Cayan, C. A. Talbot, Atmospheric rivers drive flood damages in the western United States *Sci. Adv.* **5**, eaax4631 (2019).
15. F. M. Ralph, J. J. Rutz, J. M. Cordeira, M. Dettinger, M. Anderson, D. Reynolds, L. J. Schick, C. Smallcomb, A scale to characterize the strength and impacts of atmospheric rivers *Bull. Am. Meteorol. Soc.* **100**, 269–289 (2019).
16. H. D. Prince, P. B. Gibson, M. J. De Florio, T. W. Corringham, A. Cobb, B. Guan, F. Martin Ralph, D. E. Waliser, Genesis locations of the costliest atmospheric rivers impacting the western United States. *Geophys. Res. Lett.* **48**, e2021GL093947 (2021).
17. A. M. Rhoades, M. D. Risser, D. A. Stone, M. F. Wehner, A. D. Jones, Implications of warming on western United States landfalling atmospheric rivers and their flood damages. *Weather Clim. Extremes* **32**, 100326 (2021).
18. T. W. Corringham, J. McCarthy, T. Shulgina, A. Gershunov, D. R. Cayan, F. M. Ralph, Climate change contributions to future atmospheric river flood damages in the western United States. *Sci. Rep.* **12**, 13747 (2022).
19. C. W. Hecht, J. M. Cordeira, Characterizing the influence of atmospheric river orientation and intensity on precipitation distributions over North Coastal California. *Geophys. Res. Lett.* **44**, 9048–9058 (2017).
20. H. V. Griffith, A. J. Wade, D. A. Lavers, G. Watts, Atmospheric river orientation determines flood occurrence. *Hydrol. Process.* **34**, 4547–4555 (2020).
21. Q. Cao, A. Gershunov, T. Shulgina, F. M. Ralph, N. Sun, D. P. Lettenmaier, Floods due to atmospheric rivers along the U.S. west coast: The role of antecedent soil moisture in a warming climate. *J. Hydrometeorol.* **21**, 1827–1845 (2020).
22. E. Sumargo, H. McMillan, R. Weihs, C. J. Ellis, A. M. Wilson, F. M. Ralph, A soil moisture monitoring network to assess controls on runoff generation during atmospheric river events *Hydrol. Process.* **35**, e13998 (2021).
23. E. Sumargo, F. Cannon, F. M. Ralph, B. Henn, Freezing level forecast error can consume reservoir flood control storage: Potentials for lake oroville and new bullards bar reservoirs in california. *Water Resour. Res.* **56**, e2020WR027072 (2020).
24. M. D. Warner, C. F. Mass, E. P. Salatheé, Wintertime extreme precipitation events along the pacific northwest coast: Climatology and synoptic evolution. *Mon. Weather Rev.* **140**, 2021–2043 (2012).

25. J. Jasperse, M. Ralph, M. Anderson, L. D. Brekke, M. Dillabough, M. D. Dettinger, A. Haynes, R. Hartman, C. Jones, J. Forbis, P. Rutten, C. Talbot, R. H. Webb, Preliminary viability assessment of Lake Mendocino forecast informed reservoir operations, *Tech. rep.* (2017).
26. I. Mallakpour, A. AghaKouchak, M. Sadegh, Climate-induced changes in the risk of hydrological failure of major dams in California. *Geophys. Res. Lett.* **46**, 2130–2139 (2019).
27. J. Zscheischler, O. Martius, S. Westra, E. Bevacqua, C. Raymond, R. M. Horton, B. van den Hurk, A. AghaKouchak, A. Jézéquel, M. D. Mahecha, D. Maraun, A. M. Ramos, N. N. Ridder, W. Thiery, E. Vignotto, A typology of compound weather and climate events *Nat. Rev. Earth Environ.* **1**, 333–347 (2020).
28. B. J. Moore, A. B. White, D. J. Gottas, Characteristics of long-duration heavy precipitation events along the west coast of the United States. *Mon. Weather Rev.* **149**, 2255–2277 (2021).
29. National Environmental Satellite Data and Information Service, Atmospheric Rivers Hit West Coast (2023).
30. P. Rogers, California storms: The past three weeks were the wettest in 161 years in the Bay Area (2023).
31. NOAA NCEI, U.S. Billion-Dollar Weather and Climate Disasters (2023).
32. B. Henn, K. N. Musselman, L. Lestak, F. M. Ralph, N. P. Molotch, Extreme runoff generation from atmospheric river driven snowmelt during the 2017 oroville dam spillways incident. *Geophys. Res. Lett.* **47**, e2020GL088189 (2020).
33. A. C. Michaelis, A. Gershunov, A. Weyant, M. A. Fish, T. Shulgina, F. M. Ralph, Atmospheric river precipitation enhanced by climate change: A case study of the storm that contributed to California's Oroville Dam Crisis. *Earth's Future* **10**, e2021EF002537 (2022).
34. X. Huang, D. L. Swain, Climate change is increasing the risk of a California megaflood. *Sci. Adv.* **8**, eabq0995 (2022).
35. C. Bowers, K. A. Serafin, K.-C. Tseng, J. W. Baker, Atmospheric river sequences as indicators of hydrologic hazard in historical reanalysis and GFDL SPEAR future climate projections. *Earth's Future* **11**, e2023EF003536 (2023). https://doi.org/10.1029/2023EF003536
36. M. A. Fish, A. M. Wilson, F. M. Ralph, Atmospheric river families: Definition and associated synoptic conditions. *J. Hydrometeorol.* **20**, 2091–2108 (2019).

37. M. A. Fish, J. M. Done, D. L. Swain, A. M. Wilson, A. C. Michaelis, P. B. Gibson, F. M. Ralph, Large-scale environments of successive atmospheric river events leading to compound precipitation extremes in california. *J. Climate* **35**, 1515–1536 (2022).
38. J. J. Rutz, W. J. Steenburgh, F. M. Ralph, Climatological characteristics of atmospheric rivers and their inland penetration over the western united states. *Mon. Weather Rev.* **142**, 905–921 (2014).
39. NOAA National Centers for Environmental Information (NCEI), Storm Events Database (2023).
40. FEMA, OpenFEMA Data Sets (2023).
41. M. W. Downton, J. Z. B. Miller, R. A. Pielke Jr, Reanalysis of U.S. National Weather Service Flood Loss Database. *Nat. Hazards Rev.* **6**, 13–22 (2005)
42. R. Gelaro, W. McCarty, M. J. Suárez, R. Todling, A. Molod, L. Takacs, C. A. Randles, A. Darmenov, M. G. Bosilovich, R. Reichle, K. Wargan, L. Coy, R. Cullather, C. Draper, S. Akella, V. Buchard, A. Conaty, A. M. da Silva, W. Gu, G. K. Kim, R. Koster, R. Lucchesi, D. Merkova, J. E. Nielsen, G. Partyka, S. Pawson, W. Putman, M. Rienecker, S. D. Schubert, M. Sienkiewicz, B. Zhao, The modern-era retrospective analysis for research and applications, version 2 (MERRA-2). *J. Climate* **30**, 5419–5454 (2017).
43. J. W. Baldwin, J. B. Dessy, G. A. Vecchi, M. Oppenheimer, Temporally compound heat wave events and global warming: An emerging hazard. *Earth's Future* **7**, 411–427 (2019).
44. S. D. Changnon, Measures of economic impacts of weather extremes. *Bull. Am. Meteorol. Soc.* **84**, 1231–1236 (2003).
45. M. W. Downton, R. A. Pielke, How accurate are disaster loss data? The case of U.S. flood damage *Nat. Hazards* **35**, 211–228 (2005).
46. M. Gall, K. A. Borden, S. L. Cutter, When do losses count?, *Bull. Am. Meteorol. Soc.* **90**, 799–810 (2009).
47. J. Czajkowski, G. Villarini, M. Montgomery, E. Michel-Kerjan, R. Goska, Assessing current and future freshwater flood risk from north atlantic tropical cyclones via insurance claims. *Sci. Rep.* **7**, 41609 (2017).
48. T. W. Corringham, D. R. Cayan, The effect of El Niño on flood damages in the western United States, *Clim. Soc.* **11**, 489–504 (2019).
49. J. Knighton, B. Buchanan, C. Guzman, R. Elliott, E. White, B. Rahm, Predicting flood insurance claims with hydrologic and socioeconomic demographics via machine learning: Exploring the roles of

topography, minority populations, and political dissimilarity. *J. Environ. Manage.* **272**, 111051 (2020).

50. A. Sebastian, D. J. Bader, C. M. Nederhoff, T. W. B. Leijnse, J. D. Bricker, S. G. J. Aarninkhof, Hindcast of pluvial, fluvial, and coastal flood damage in Houston, Texas during hurricane Harvey (2017) using SFINCS. *Nat. Hazards* **109**, 2343–2362 (2021).
51. A. M. Young, K. T. Skelly, J. M. Cordeira, High-impact hydrologic events and atmospheric rivers in California: An investigation using the NCEI Storm Events Database. *Geophys. Res. Lett.* **44**, 3393–3401 (2017).
52. E. Dougherty, K. L. Rasmussen, Climatology of flood-producing storms and their associated rainfall characteristics in the United States. *Mon. Weather Rev.* **147**, 3861–3877 (2019).
53. F. V. Davenport, M. Burke, N. S. Diffenbaugh, Contribution of historical precipitation change to US flood damages. *Proc. Natl. Acad. Sci. U.S.A.* **118**, e2017524118 (2021).
54. M. Williamson, K. Ash, M. J. Erickson, E. Mullens, Damages associated with excessive rainfall outlooks (ERO) and missed flash floods *Weather Forecast.* **38**, 971–984 (2023).
55. N. Japkowicz, AAAI Workshop on Learning from Imbalanced Data Sets (2000).
56. T. Hasanin, T. Khoshgoftaar, The Effects of Random Undersampling with Simulated Class Imbalance for Big Data. Paper Presented at the 2018 IEEE International Conference on Information Reuse and Integration (IRI), Salt Lake City, UT, USA, 6 to 9 July 2018.
57. E. E. Butler, P. Huybers, Adaptation of US maize to temperature variations. *Nat. Clim. Chang.* **3**, 68–72 (2013).
58. S. Heft-Neal, J. Burney, E. Bendavid, M. Burke, *Nature* **559**, 254 (2018), 258, Robust relationship between air quality and infant mortality in Africa.
59. M. Hino, S. T. Belanger, C. B. Field, A. R. Davies, K. J. Mach, High-tide flooding disrupts local economic activity. *Sci. Adv.* **5**, eaau2736 (2019).
60. J. C. Timoneda, Estimating group fixed effects in panel data with a binary dependent variable: How the LPM outperforms logistic regression in rare events data *Soc. Sci. Res.* **93**, 102486 (2021).
61. C. Bowers, Supplemental Code Release: Atmospheric River Sequences as Indicators of Hydrologic Hazard in Present and Future Climates Collection. (2023).
62. C. A. Shields, J. J. Rutz, L. Y. Leung, F. M. Ralph, M. Wehner, B. Kawzenuk, J. M. Lora, E. McClenny, T. Osborne, A. E. Payne, P. Ullrich, A. Gershunov, N. Goldenson, B. Guan, Y. Qian, A. M. Ramos, C. Sarangi, S. Sellars, I. Gorodetskaya, K. Kashinath, V. Kurlin, K. Mahoney, G.

Muszynski, R. Pierce, A. C. Subramanian, R. Tome, D. Waliser, D. Walton, G. Wick, A. Wilson, D. Lavers, Mr. Prabhat, A. Collow, H. Krishnan, G. Magnusdottir, P. Nguyen, Atmospheric River Tracking Method Intercomparison Project (ARTMIP): Project goals and experimental design. *Geosci. Model Dev.* **11**, 2455–2474 (2018).

63. C. A. Shields, ARTMIP Tier1 Catalogues (2023).
